# Impact of Genetic Variants of Apolipoprotein E on Lipid Profile in Patients with Parkinson's Disease

**DOI:** 10.1155/2013/641515

**Published:** 2013-09-24

**Authors:** Michele L. Gregório, Marcela A. S. Pinhel, Caroline L. Sado, Gabriela S. Longo, Fábio N. Oliveira, Gisele S. Amorim, Marcelo A. Nakazone, Greiciane M. Florim, Camila M. Mazeti, Denise P. Martins, Waldir A. Tognola, Antonio C. Brandão, Sidney Pinheiro Júnior, Moacir F. de Godoy, Dorotéia R. S. Souza

**Affiliations:** ^1^Department of Molecular Biology, Sao Jose do Rio Preto Medical School (FAMERP), 15090000 Sao Jose do Rio Preto, SP, Brazil; ^2^Sao Jose do Rio Preto Medical School (FAMERP), Center of Research in Biochemistry and Molecular Biology, NPBIM, Avenida Brigadeiro Faria Lima 5416, Vila Sao Pedro, 15090000 Sao Jose do Rio Preto, SP, Brazil; ^3^Federal University of São Paulo (UNIFESP), 04021001 São Paulo, SP, Brazil; ^4^Hospital de Base, 15090000 Sao Jose do Rio Preto, SP, Brazil; ^5^Sao Jose do Rio Preto Medical School (FAMERP), 15090000 Sao Jose do Rio Preto, SP, Brazil; ^6^Department of Neuroscience/Molecular Biology, Sao Jose do Rio Preto Medical School (FAMERP), 15090000 Sao Jose do Rio Preto, SP, Brazil; ^7^Department of Molecular Biology, Sao Jose do Rio Preto Medical School—FAMERP, 15090000 Sao Jose do Rio Preto, SP, Brazil

## Abstract

The pathogenesis of Parkinson's disease (PD) seems to involve genetic susceptibility to neurodegeneration. *APOE* gene has been considered a risk factor for PD. This study aimed to evaluate the association of *APOE* polymorphism with PD and its influence on lipid profile. We studied 232 PD patients (PD) and 169 individuals without the disease. The studied polymorphism was analyzed by PCR/RFLP. The Fisher's exact test, chi-square, ANOVA, and *t*-test (*P* < 0.05) were applied. The *APOE3/3* genotype was prevalent in PD patients and Controls (*P* = 0.713) followed by *APOE3/4* (*P* = 0.772). Both groups showed recommended values for lipid profile, with increase in the values of total cholesterol and LDLc, as well as decreased values of triglycerides in PD patients compared with Controls (*P* < 0.05 for all of them). Increased levels of HDLc, in PD patients, were associated with the *APOE3/3* versus *APOE-/4* genotypes (*P* = 0.012). The *APOE *polymorphism does not distinguish PD patients from Controls, as opposed to the lipid profile alone or in association with *APOE*. Furthermore, a relationship between increase of HDLc levels and *APOE3* in homozygous was found in PD patients only.

## 1. Introduction

Parkinson's disease (PD) is a complex neurodegenerative disorder, chronic and progressive, affecting 2% of the population older than 65 years [1]. The identification of specific biomarkers for PD is one of the main goals of this clinical research. Today the diagnosis of this second most common neurological disease is possible by clinical evaluation of extrapyramidal signs, such as tremor, rigidity, and bradykinesia. These symptoms occur when the degeneration of nigral dopaminergic neurons of substantia nigra (SN), which project to the striatum [1], disrupting the motor circuit of the basal ganglia, has risen over 70% [2–4]. It has been suggested that cognitive deficit is a common feature of PD [1].

In Brazil, the incidence of PD is equivalent to 150/200 cases per 100,000 inhabitants, with the emergence of 20/100,000 new cases per year [5]. The etiology of PD seems to involve genetic susceptibility and environmental factors [6]. Some evidence shows that mitochondrial dysfunction, oxidative stress, and genetic factors play an important role in the pathogenesis of this disease [7]. Approximately 85% of PD cases are sporadic, 10–15% of patients have family history, and less than 5% of them present monogenic inheritance [8].

The apolipoprotein E (ApoE) plays an important role in the lipoprotein metabolism [9] and transport of lipids to tissues [10]. It serves as a ligand for at least two specific receptors of lipoproteins, the low density lipoprotein receptor (LDLr) or ApoB/E and the liver receptor to apoE, the lipoprotein receptor-related proteins (LRP = LDL receptor related protein), allowing the removal of these particles by the liver [11]. The apolipoprotein E (ApoE) is also the main apolipoprotein in the central nervous system, with evidence of its association with cerebrovascular diseases [12], and neurodegenerative diseases as late onset of Alzheimer's Disease (AD) [13, 14] and PD [15, 16]. Thus, *APOE* gene has become a significant target for investigation in neurodegenerative diseases.

The *APOE* gene, containing four exons, is mapped on the human chromosome 19q13.2 [17]. The *APOE* polymorphism, located on exon 4, is identified in the form of three major alleles *APOE2 *(prevalence of 0.01 to 0.15), *APOE3* (0.49 to 0.91), and *APOE4* (0.06 to 0.37) [18], which determines three protein isoforms (E2, E3, and E4, resp.) and six possible genotypes (e2/e2, e2/e3, e2/e4, e3/e3, e3/e4, and e4/e4). E2 and E3 clear plaques 20 times more efficiently than E4 [19]. E3 seems to be the normal isoform in all known functions, while E4 and E2 can each be dysfunctional [19]. Some researches and a meta-analysis study have shown that the e2 allele is associated with higher risk of PD development [18, 20, 21], whereas other studies have shown that the e4 allele is responsible for PD development [15, 21–23]. Therefore, both the e2 and the e4 alleles may play a role in PD development. APOE is responsible for clearance of the b-amyloid plaques which impair the nervous system [24]. Therefore, the *APOE4* allele is associated with high concentrations of LDLc and *APOE2* at low plasma levels of LDLc [25]. The apoE is synthesized by astrocytes within the brain, and among its polymorphisms, the *APOE4* allele, in particular, seems to have a risk effect on PD and a possible relationship with the existing neurodegeneration among Parkinson's disease patients. Some studies have considered *ε*4 as a risk factor for the age of onset for PD, decline in cognitive impairment, and/or development of dementia in PD. *APOE2* has also been identified as an important risk factor for PD, however, with weak and inconsistent effect across studies [1, 26–34].

Therefore, the change in lipid metabolism mediated by different isoforms of apoE could influence the neuronal degenerative processes. Several mechanisms have been proposed associating increased risk of PD development with higher plasma levels of total cholesterol (TC) [35]. However, only a few case-control studies [36, 37] and a few prospective epidemiological findings [38, 39] evaluated such association but with no conclusive results. In this case, prospective studies showing reduced risk of PD with increased plasma levels of TC were highlighted [35, 38].

In accordance with the multifactorial etiologic hypothesis for PD susceptibility genes, even though modest, may have an association with the disease [18]. Thus, this study aimed to evaluate the association among polymorphisms of *APOE *and PD, and to provide a comparative analysis of lipid profile in different ApoE genotypes in patients with PD and individuals without the disease.

## 2. Methods

This is a randomized case-control study performed with individuals (*N* = 401) with mixed ethnicity [40]. They were divided into PD patients: 232 patients with PD, mean age = 69.2 ± 11.1 years and 62% male and Controls: 169 individuals not diagnosed with PD, mean age = 71.7 ± 8.5 years and 48% male. Patients were selected at the Movement Disorders Clinic of the Hospital de Base of Sao Jose do Rio Preto Medical School, SP, Brazil. The diagnosis of PD was performed by a neurologist specializing in movement disorders, who applied the clinical criteria recommended by Jankovic [2], including bradykinesia, rigidity, tremor at rest, postural instability, unilateral onset, response to L-dopa for more than 5 years, levodopa-induced dyskinesia, progressive disorder, persistent asymmetry and clinical course of 10 years or more, and complementary tests [2]. In the diagnosis of PD, we included at least one neuroimaging method (skull PET, MRI, or cerebral scintigraphy by SPECT). Healthy individuals (Controls) were treated by other specialists of the same treatment unit and had the same age as those patients selected in the PD group. Those individuals under hypolipidemic drug therapy and/or any specific diet were excluded from both groups. All subjects were informed about the nature of the study and confirmed their willingness to participate by signing written consent forms. This study was approved by the Ethics Research Committee of the institution (opinion/no. 151/2008—Certificate of Appreciation Presentation Ethics (CAAE) 0029.0.140.000-08). 

Peripheral blood was collected in order to obtain analysis of genetic polymorphisms for *APOE* and lipid profile, including plasma levels of total cholesterol (TC), low density lipoprotein cholesterol (LDLc), high density lipoprotein cholesterol (HDLc) and very low density (VLDLc), and triglycerides (TG). Lipid profile tests were partially done for the PD patients (*N* = 86) as it was difficult to collect enough biological material (peripheral blood) for all analyses (genetic and biochemical). The study of *APOE* polymorphism (rs429358 and rs7412) was performed in the Center for Research in Biochemistry and Molecular Biology of Sao Jose do Rio Preto Medical School and consisted of genomic DNA extraction from whole blood samples (5 mL) [41] and DNA amplification by conventional PCR (polymerase chain reaction) and enzymatic restriction (*Hha* I). 

Each reaction was performed in Eppendorf-Mastercycler thermocycler; each tube contained 0.5 *μ*L of nucleotides (0.8 mM), 2.5 *μ*L of buffer PCR 10X, 2.5 *μ*L of dimethyl sulfoxide 10%, 2.5 *μ*L of each primer (2.5 *μ*M), 0.2 *μ*L of Taq polymerase (5 U/*μ*L), 11 *μ*L of Milli Q water, and 2 *μ*L of diluted genomic DNA (0.2 *μ*g). Complementary primers were used in regions next to the polymorphic codons 112 and 158, located in exon 4 of *APOE* (rs429358 and rs7412) P1: 5′-ACA GAA TTC GCC CGG CCT GGT ACA C-3′ and P2: 5′-TAA GCT TGG CAC GGC TGT CCA AGC A-3′ [42]. Initial DNA denaturation was obtained at 94°C during 5 minutes, and the reaction mix submitted to 40 cycles of 94°C during 30 seconds and 65°C during 2 minutes, extension at 72°C during 1 minute, and ending cycle at 72°C during 7 minutes [42].

The analysis of ApoE genetic variants was performed by restriction fragment length polymorphism (RFLP) analysis, with the restriction enzyme* Hha *I *fast* (5 U per reaction tube) in double boiler at 37°C during 45 minutes, for cleavage of amplified sequences in specific regions (GCGC), identifying the alleles *APOE2*, *APOE3*, and *APOE4*. The DNA fragments were separated by electrophoresis on 6% polyacrylamide gel nondenatured, under constant electric current of 180 V during 2 hours. A standard DNA sample (100 base pairs, Invitrogen) was used as comparison with the electrophoretic bands of patients. The gel was colored by GelRed (Uniscience of Brazil) during 10 minutes, and DNA fragments were visualized under ultraviolet light (UV).

## 3. Statistical Analysis

Categorical variables (including demographic data and genetic variants) were analyzed applying Fisher's exact test and the Chi-square test. Continuous variables (age, values for lipid profile) were analyzed by ANOVA, Tukey, *t*-test, or Mann-Whitney test. *P* < 0.05 was considered statistically significant. Statistical analysis also included Hardy-Weinberg equilibrium (Fisher's exact test and the Chi-square test).

## 4. Results 

Table 1 shows the genotype distribution and allele frequencies of the *APOE* polymorphism. We observed the prevalence of *APOE3/3* genotype in PD patients (73.0%) and Controls (75.2%, *P* = 0.713), followed by *APOE3/4 *(15.9%, 17.5%, resp.; *P* = 0.772). There was a similar distribution of alleles of the ApoE in both groups (*P* > 0.05). 

 Concerning the lipid profile (Table 2), both groups showed values within the reference limits of normality, except for patients with slightly increased TC mean value (202.5 ± 45.7 mg/dL). This group showed higher TC and LDLc levels (122.0 ± 38.7 mg/dL) and lower VLDLc and TG levels (22.8 ± 13.3 mg/dL, 115.5 ± 66.7 mg/dL, resp.), compared with Controls (186.8 ± 51.1 mg/dL; *P* = 0.017; 102.8 ± 44.9 mg/dL; *P* = 0.001; 31.3 ± 29.4 mg/dL; *P* = 0.001; 136.9 ± 73.5 mg/dL; *P* = 0.024, resp.). 

Table 3 shows the relationship between lipid profile and *APOE* polymorphism. PD patients present a relationship between *APOE-/4* and a decrease in HDLc levels (51.8 ± 10.5 mg/dL), compared with *APOE3/3* (60.3 ± 13.3 mg/dL; *P* = 0.025). Furthermore, patients with *APOE3/3* genotype showed significantly higher levels of HDLc (60.3 ± 13.3 mg/dL) and reduced levels of VLDLc and TG (22.7 ± 13.1 mg/dL; 113 ± 65.7 mg/dL, resp.), compared with Controls with the same genotype (52.0 ± 15.5 mg/dL; *P* = 0.001; 36.6 ± 37.4 mg/dL; *P* = 0.001; 143.5 ± 79.8 mg/dL; *P* = 0.017, resp.). In Controls, individuals with *APOE3/3 *genotype presented higher values of VLDLc, compared with those with *APOE-/4* genotypes (*P* = 0.002).

## 5. Discussion

This study shows similar genotype distribution and allele frequency for the *APOE* polymorphism in PD patients and Controls, as described by other authors [1, 25–28]. On the other hand, a meta-analysis study highlights the increased susceptibility to the disease associated with presence of the *APOE2* allele (odds ratio: 1.16; confidence interval 95%: 1.03–1.31) [43], however, not observed in this study. Differences among studies may be explained by changes in methodology, sample size, age of manifestation of the disease, ethnicity, and other environmental and geographic factors which can be considered in the analysis of genetic polymorphisms. 

*ApoE* genotype distribution differs among populations. Eichner et al. [44] reviewed the frequencies of E2, E3, and E4 alleles in different populations and reported that these range from 0.02 to 0.13 for E2, 0.06 to 0.85 for E3, and 0.11 to 0.31 for E4. The prevalence of the E4 allele in Brazil is similar to that observed in other South American countries with frequencies between 0.23 and 0.26 [14], except for Chileans with a frequency of 0.40. However, in Controls, the prevalence ranges between 0.08 and 0.19 [45]. These frequencies for the E4 allele are lower than in populations from the northern hemisphere, whose frequencies vary between 0.38 and 0.48 [46, 47].

The possible association of apoE and risk of PD has been widely investigated in different populations. However, it remains underrepresented in the Brazilian population. Thus, this is a pioneer study in terms of the distribution of ApoE genetic polymorphisms and its relationship with lipid profiles in patients with PD, helping understand possible genetic markers for this disease. Considering the relationship of apoE and lipid metabolism [48], we also evaluated the lipid profile. The mean values were within the reference limits except for TC, with slight increase in PD patients [49], who also showed increased levels of LDLc and reduction in VLDLc and TG levels, compared with Controls.

 Clinical and subclinical conditions [50, 51] can be associated with reduced concentrations of cholesterol (TC, LDLc and HDLc), as observed in inflammatory diseases [50, 52]. Studies also show changes in lipid metabolism associated with neurodegenerative diseases, including Alzheimer's disease and PD [53, 54]. In this case, there is reference to reduction in the synthesis of cholesterol in skin fibroblasts from patients with PD [55], as well as low values of TC compared with Controls [35]. 

In an analysis stratified by gender, Pena et al. [40] observed a reduction in the risk of PD with increased levels of TC only in women. In men, only higher values of TC accounted for reduced risk for PD, compared with men with lower values of TC. However, de Lau et al. [38] found no relationship among LDLc concentration, duration of PD, and use of dopaminergic agents. Therefore, both authors suggest that the relationship between low levels of LDLc and PD is a consequence of the disease or its treatment. 

Additionally, there is speculation that high levels of TC raise the risk of PD [36], partly due to the excess of body weight, as some studies observed a relationship between excess weight and a higher risk for PD [36, 56]. Also, there is an inverse association between intake of total fat and unsaturated fatty acids with risk of PD [57, 58]. Thus, a number of factors strongly influence the variation of lipid profile in PD patients, which requires strict criteria to be applied on the selection of the studied groups. 

This study also highlights the clear influence of apoE polymorphisms and lipid profile between the groups. In this case, PD patients showed increased HDLc levels, compared with Controls, only in those patients with *APOE3/3 *genotype, who also showed reduced levels of TG and VLDLc when compared with Controls. Additionally, only PD patients showed the relationship between the presence of *APOE4* and reduced serum levels of HDLc, compared with *APOE3/3* genotype. Thus, PD patients and Controls show differences in the combination of lipid profile and the apoE-*Hha* I polymorphism. On the other hand, Huang et al. [18] detected increased risk of PD, associated with reduced plasma levels of TC related to the allele *APOE2. *Thus, these controversies may reflect changes in lifestyle habits, drug treatment, and medical advice to control the disease, which are aspects to be investigated in further studies.

Therefore, this study allows us to conclude that *APOE* polymorphism does not distinguish PD patients from Controls, as opposed to the lipid profile alone or in association with *APOE*. In this case, and increase in TC and LDLc levels can be observed in PD patients, whereas higher VLDLc and TG levels are prevalent in Controls. Moreover, only PD patients show a relationship between increase of HDLc levels and *APOE3* in homozygous. Further studies including subgroups of patients with and without family history of PD are needed to clarify the influence of genetic polymorphisms and their respective mechanisms in PD.

## Figures and Tables

**Table 1:** Distribution of absolute and relative frequencies of alleles and genotypes of *APOE* polymorphism in individuals with Parkinson's disease (PD) and without (Controls).

Genotype *APOE *	PD (*N* = 232)		Controls (*N* = 137)		P*
	*N *	%	*N *	%	
Genotype					
*APOE2/4 *	1	0.4	0	—	—
*APOE2/3 *	19	8.1	8	5.8	0.535
*APOE3/3 *	169	73.0	103	75.2	0.713
*APOE3/4 *	37	15.9	24	17.5	0.772
*APOE4/4 *	6	2.6	2	1.5	0.727
** **Total	**232**	**100**	**137**	**100**	
*APOE-/4 *	44	18.9	26	18.9	1.000
*APOE-/2 *	20	8.6	8	5.8	0.413
Allele					
*APOE2 *	20	0.04	8	0.03	0.426
*APOE3 *	394	0.85	238	0.87	0.515
*APOE4 *	50	0.11	28	0.10	0.901
** **Total	**464**	**1.0**	**274**	**1.0**	

*Fisher or Chi-squared tests; *N*: total number of individuals; Abs. Freq.: absolute frequency.

**Table 2:** Distribution of means and standard deviation for lipid profile in patients with Parkinson's disease (PD) and without (Controls).

Biochemical profile mg/dL	PD (*N* = 86)	Controls (*N* = 169)	P*
TC			
Mean	202.5	186.8	**0.017**
SD	45.7	51.1	
HDLc			
Mean	58.0	56.8	0.574
SD	13.2	20.9	
LDLc			
Mean	122.0	102.8	**0.001**
SD	38.7	44.9	
VLDLc			
Mean	22.8	31.3	**0.001**
SD	13.3	29.4	
TG			
Mean	115.5	136.9	**0.024**
SD	66.7	73.5	

*t-test; TC: total cholesterol; HDLc: high density lipoprotein cholesterol; LDLc: low density lipoprotein cholesterol; VLDLc: very low density lipoprotein cholesterol; TG: triglycerides; SD: standard deviation; *N*: number of individuals.

**Table 3:** Means and standard deviations for lipid profile in patients with Parkinson's disease (PD) and without (Controls) considering *APOE* polymorphism.

Biochemical profile (mg/dL)	PD (*N* = 232)		Controls (*N* = 137)		*P *
	*APOE-/4* (a) (*N* = 15)	*APOE3/3* (b) (*N* = 58)	*APOE-/4* (c) (*N* = 19)	*APOE3/3* (d) (*N* = 94)	
TC					
Mean	206.5	203.7	200.7	190.4	0.391*
SD	48.7	47.4	59.4	54.5	
LDLc					
Mean	128.7	121.7	123.1	109.9	0.254*
SD	43.3	38.8	55.0	46.1	
HLDc					
Mean	51.8	60.3	56.5	52.0	0.007*
SD	10.5	13.3	15.9	15.5	(axb *P* = 0.012; bxd *P* = 0.001)^†^
VLDLc					
Mean	24.3	22.7	22.6	36.3	0.024*
SD	17.1	13.1	9.0	37.4	(cxd *P* = 0.001)^†^
TG					
Mean	126.0	113.3	129.7	143.5	0.039*
SD	82.5	65.7	83.5	79.8	(bxd *P* = 0.010)^†^

*One-Way ANOVA Test; ^†^ *t*-test; TC: total cholesterol; HDLc: high density lipoprotein cholesterol; LDLc: low density lipoprotein cholesterol; VLDLc: very low density lipoprotein cholesterol; TG: triglycerides; SD: standard deviation; *N*: number of individuals.
